# Host plant influences on the developmental and reproductive biology of *Aleurodicus dispersus* and its implications for ecological pest management

**DOI:** 10.1038/s41598-026-47835-6

**Published:** 2026-04-06

**Authors:** T. Boopathi, N. Anusha, J. G. Prasuna

**Affiliations:** 1 Department of Agricultural Entomology, Tamil Nadu AgriculturalUniversity, Coimbatore, Tamil Nadu 641003 India; 2 ICAR-Indian Institute of Oilseeds Research, Rajendranagar, Hyderabad, Telangana 500030 India

**Keywords:** *Aleurodicus dispersus*, Host plants, Developmental biology, Fecundity, Integrated pest management, Zoology, Entomology

## Abstract

The spiraling whitefly, *Aleurodicus dispersus* Russell (Hemiptera: Aleyrodidae), is a highly polyphagous pest that poses a major threat to tropical and subtropical agriculture. Its rapid proliferation across multiple crop species is influenced by host plant quality, which significantly affects its development, reproduction, and survival. This study evaluated the influence of ten different host plants (cassava, cotton, guava, papaya, banana, mulberry, chilli (*Capsicum annuum*), *Acalypha indica*, eggplant, and Indian beech) on the developmental and reproductive parameters of *A. dispersus* under insect-proof screen house conditions. Developmental and reproductive traits varied significantly among host plants. The total developmental period (TDP) ranged from 24.2 ± 2.39 days on mulberry to 31.9 ± 3.35 days on banana (*F* = 6.06; df = 9, 90; *P* < 0.001). Egg hatchability and adult emergence were highest on cotton (98.7% and 98.2%, respectively) and lowest on Indian beech and eggplant. Fecundity was highest on cassava (28.5 ± 3.72 eggs per female) and lowest on eggplant (13.1 ± 1.79 eggs per female) (*F* = 27.20; df = 9, 90; *P* < 0.001). Female longevity was longest on cotton and cassava. A strong positive correlation was observed between egg hatchability and adult emergence (*r* = 0.85; *P* < 0.001), whereas a significant negative correlation was found between TDP and fecundity (*r* = − 0.78; *P* < 0.001). These results demonstrates host plant species significantly influence the biological performance of *A. dispersus*. Cassava, cotton, and guava supported comparatively faster development and higher reproductive output, indicating their suitability for population buildup. The findings provide useful insights for host-based pest management and highlight the potential role of less suitable host plants in reducing whitefly population growth within integrated pest management (IPM) programs.

The spiraling whitefly, *Aleurodicus dispersus* Russell (Hemiptera: Aleyrodidae), is a highly invasive and polyphagous insect pest that poses serious threats to agricultural and horticultural crops in tropical and subtropical regions worldwide. Native to Central and South America, *A. dispersus* has successfully expanded its geographical distribution across Asia, Africa, and the Pacific, infesting a wide range of host plants including fruits, vegetables, ornamentals, and forest trees^1–3^. In India, the pest has spread rapidly since its introduction, infesting economically important crops such as cassava, papaya, guava, banana, and mulberry, and causing considerable yield and quality losses.

Damage caused by *A. dispersus* occurs through both direct and indirect mechanisms. Nymphs and adults feed on plant phloem sap, resulting in chlorosis, leaf curling, wilting, and premature leaf drop. Throughout its life cycle, the insect undergoes successive moults, and its development is heavily influenced by the nutritional quality of the host. In addition, the excretion of large quantities of honeydew promotes the growth of sooty mould, which significantly reduces photosynthetic efficiency and the commercial value of fruits and foliage^4^. Although *A. dispersus* is not known to transmit plant viruses, its rapid population growth, wide host range, and favour for physiological plant processes make it an economically important pest in many cropping systems^5^.

Insect-plant interactions, particularly host plant selection, play a critical role in determining herbivore performance and population dynamics. Host plants can directly influence insect development, reproduction and survival through factors such as nutritional composition, secondary metabolites, leaf surface characteristics, and trichome density^6,7^. For phloem-feeding insects such as whiteflies, these plant attributes can substantially alter life history traits including developmental duration, egg viability, fecundity and adult longevity^8^. Specifically, a host plant that provides a favourable environment will result in higher egg hatchability and shorter nymphal stages. Several studies have demonstrated that *Bemisia tabaci* (Gennadius), a closely related whitefly species, exhibits significant variation in developmental rate and reproductive performance depending on host plant species, with improved performance observed on hosts such as cotton and tomato^9,10^.

Although previous studies have examined the biology of *A. dispersus* on individual host plants such as cassava, guava, and papaya^11,12^, comparative investigations evaluating multiple life history traits across a diverse range of host plants including cotton, mulberry, and chilli under insect-proof screen house conditions remain limited. Furthermore, relationships between key developmental and reproductive parameters have rarely been quantified, particularly the association between egg hatchability and adult emergence or between developmental duration and fecundity. Understanding these correlations is essential for identifying which host species favour rapid population outbreaks. Such relationships are important for understanding population growth potential and improving pest risk forecasting models. Considering the continued spread of *A. dispersus* into new agroecological regions and its ability to exploit multiple host plants, a systematic evaluation of its biological performance across important crop species is necessary.

To address these gaps, the present study examined the developmental and reproductive biology of *A. dispersus* on ten commonly host plants under insect-proof screen house conditions. The study utilized a robust observational design to track 100 individuals per host, ensuring that stage-specific mortality and success rates were accurately captured. Biological parameters including egg period, duration of nymphal instars, pupal period, total developmental period (TDP), egg hatchability, adult emergence, fecundity, adult longevity, and sex ratio were recorded. In addition, relationships between critical life history traits, specifically total developmental period and fecundity as well as egg hatchability and adult emergence, were analyzed to better understand how host plants influence pest population dynamics.

We hypothesized that host plant species would significantly influence the developmental and reproductive performance of *A. dispersus*. Host plants with higher nutritional quality and softer leaf tissues, such as cassava and papaya, were expected to represent a favourable environment that supports faster development and greater reproductive output. In contrast, host plants with tougher leaves or stronger chemical defenses, such as Indian beech and banana, were expected to result in prolonged developmental duration and reduced fecundity. The findings of this study are expected to provide useful insights for host plant resistance screening and for developing ecologically based pest management strategies, including the design of cropping systems that may help suppress *A. dispersus* populations naturally.

## Methods

### Study design

The study was conducted to evaluate the impact of different host plants on the developmental biology of *A. dispersus*. The research was performed under insect-proof screen house conditions with temperatures ranging from 26 to 33 °C and relative humidity maintained between 75 and 85%. This 7 °C temperature range was selected to represent the natural diurnal fluctuations of tropical and subtropical regions where the pest commonly occurs. By simulating these fluctuating conditions rather than using a constant-temperature growth chamber, the study provides a more ecologically relevant assessment of how host plants influence the pest’s physiology under field-realistic thermal stress. Temperature and relative humidity were monitored daily using a digital thermo-hygrometer to ensure all host plants were maintained under identical environmental conditions to avoid treatment bias.

### Host plant selection

Ten host plant species commonly infested by *A. dispersus* were selected based on their economic importance and field observations (Table 1): cassava (*Manihot esculenta*), papaya (*Carica papaya*), banana (*Musa* spp.), guava (*Psidium guajava*), cotton (*Gossypium hirsutum*), eggplant (*Solanum melongena*), Indian beech (*Pongamia pinnata*), chilli (*Capsicum annuum*), mulberry (*Morus alba*), and Acalypha (*Acalypha indica)*. These plants were chosen due to their significance as feeding and breeding hosts for *A. dispersus*.

**Table 1:** Details of host plants used for developmental studies of *Aleurodicus dispersus*.

Host species^#^	Scientific name	Name of the cultivar	Category	Collection
Cassava	*Manihot esculenta* Crantz.	Mulluvadi-1	Tuber crop	TNAU, Coimbatore, Tamil Nadu, India
Eggplant	*Solanum melongena* L.	Pusa Purple Round	Vegetable crop	ICAR-IARI, New Delhi, India
Chilli	*Capsicum annuum* L.	TNAU PKM-1	Vegetable crop	TNAU, Coimbatore, Tamil Nadu, India
Mulberry	*Morus alba* L.	MR2	Commercial crop	TNAU, Coimbatore, Tamil Nadu, India
Cotton	*Gossypium hirsutum* L.	CO-17	Commercial crop	TNAU, Coimbatore, Tamil Nadu, India
Guava	*Psidium guajava* L.	GUAVA-TRY 1	Fruit crop	TNAU, Coimbatore, Tamil Nadu, India
Papaya	*Carica papaya* L.	CO-8	Fruit crop	ICAR-IARI, New Delhi, India
Banana	*Musa × paradisiaca* L.	Robusta	Fruit crop	TNAU, Coimbatore, Tamil Nadu, India
Acalypha	*Acalypha indica* L.	Local	Weed	TNAU, Coimbatore, Tamil Nadu, India
Indian beech	*Millettia pinnata* (L.) Panigrahi	Local	Oil-bearing tree	TNAU, Coimbatore, Tamil Nadu, India

^#^No special permission was required for handling these plant species for the present study.

The propagation method was standardized according to species-specific requirements: cotton, chilli, eggplant, and Acalypha were established by sowing seeds, while cassava and mulberry were propagated using semi-hardwood stem cuttings. Papaya, guava and Indian beech were raised from uniform nursery seedlings, and banana was propagated via healthy sword suckers. All plants were established in sterilized soil within clay pots (30 cm diameter × 45 cm height). To ensure uniformity across treatments, all host plants were maintained in an insect-proof screen house under identical irrigation and fertilizer regimes. Only plants at the same phenological stage, possessing 4–5 fully developed leaves, were selected for the experiments to minimize variation due to plant age or biomass.

### Insect culture

A stock culture of *A. dispersus* was established from naturally infested cassava plants collected from a field in Coimbatore, Tamil Nadu, India, and maintained under insect-proof screen house conditions. Adult whiteflies were transferred onto healthy pesticide-free cassava plants to initiate the colony. While cassava was used for the initial stock culture due to its high suitability for rapid population multiplication, the colony was maintained for two successive generations prior to the experiments to ensure a uniform, healthy, and acclimatized insect population for all subsequent host plant trials.

### Experimental setup

The experiment was conducted using a Completely Randomized Design (CRD). The replication structure was organized at two levels to ensure both biological and statistical rigor: (1) Three replications were maintained for each host plant species, where each replication consisted of ten potted plants (totaling 30 plants per host); (2) From these plants, 100 individual insects per host were followed for detailed biological observations. Plants were individually covered with micro-cages (25 cm diameter × 125 cm height) made of mylar film with a muslin cloth window for aeration. For infestation, ten newly emerged adult whiteflies (five males and five females) were released onto each plant and allowed to oviposit for 24 h. After the oviposition period, adults were removed and eggs laid on the leaves were marked and monitored individually.

### Observations

To provide a clear distinction between experimental units, observations were recorded from 100 individuals per host plant species (10 individuals monitored across 10 plants). This large sample size (*n* = 100) was utilized to provide a robust dataset for each biological parameter.

The duration of the egg stage, defined as the time from oviposition to hatching, was observed daily under a stereomicroscope. After hatching, the duration of each nymphal instar (first to fourth) was individually monitored by tagging and tracking representative individuals on each host plant. The pupal period, representing the time from the last moult to adult emergence, was also recorded. These values were then used to calculate the total developmental period (TDP).

Egg hatchability was calculated as the percentage of eggs that successfully hatched, with unhatched eggs and subsequent nymphal mortality recorded at every stage to provide a complete mortality profile. Adult emergence was calculated as the percentage of pupae that successfully emerged as adults. For adults, female longevity, pre-oviposition period, and total fecundity were recorded. The sex ratio of the progeny was determined upon emergence and expressed specifically as a female: male ratio.

### Statistical analysis

Data were subjected to one-way analysis of variance (ANOVA) using SPSS software (Version 26.0, IBM Corporation, Armonk, NY, USA). All mean values are reported with their respective Standard Errors (± SE) to indicate data precision. Effect sizes were calculated using eta-squared (η^2^).

Prior to ANOVA, assumptions of normality and homogeneity of variance were verified using the Shapiro–Wilk and Levene’s tests. Treatment means were compared using Tukey’s Honest Significant Difference (HSD) post hoc test at *P* ≤ 0.01, and significant differences were denoted using superscripted alphabets in the results tables. Correlation and regression analyses (*P* ≤ 0.05) were performed to visualize the relationships between egg hatchability and adult emergence, and TDP and fecundity. For the sex ratio, Pearson’s Chi-Square test, along with Phi and Cramer’s V statistics, was used to determine the strength of association between host plants and progeny sex distribution.

## Results

### Developmental period across host plants

The developmental period of *A. dispersus* varied significantly across different host plants (Table 2). The incubation period showed significant variation among host plants (*F* = 3.16; df = 9, 90; *P* = 0.002**; η^2^ = 0.240). The highest egg period was observed on Indian beech (7.7 ± 1.34 days), while the lowest was recorded on cassava (5.5 ± 1.08 days). The 1st instar period varied significantly across host plants (*F* = 14.01; df = 9, 90; *P* < 0.001; η^2^ = 0.584). The longest duration was recorded on papaya (6.5 ± 1.27 days), whereas the shortest was on Acalypha (2.3 ± 0.48 days). The 2nd instar period also differed significantly among hosts (*F* = 22.62; df = 9, 90; *P* < 0.001; η^2^ = 0.694). The maximum duration was observed on guava (5.4 ± 0.70 days), while the minimum was on mulberry (2.2 ± 0.42 days). A significant difference was also noted in the 3rd instar period (*F* = 6.11; df = 9, 90; *P* < 0.001; η^2^ = 0.379), with the longest recorded on banana (6.1 ± 0.99 days) and the shortest on mulberry (3.2 ± 0.79 days). In contrast, the 4th instar period did not vary significantly among host plants (*F* = 1.47; df = 9, 90; *P* = 0.171ns; η^2^ = 0.128).

**Table 2:** Developmental period (days, mean ± standard deviation) of *Aleurodicus dispersus* across different host plants (*n* = 100 individuals total; *n* = 10 individuals per biological replicate).

Host species	Mean ± standard deviation^#^						
	Egg period	Nymphal period					Pupal period
		1st instar	2nd instar	3rd instar	4th instar	1st–4th instars	
Cassava	5.50 ± 1.08^b^	4.10 ± 0.74^bc^	5.10 ± 0.88^abc^	3.80 ± 0.92^b^	6.30 ± 0.95^a^	19.30 ± 1.70^abc^	2.4 ± 0.52^b^
Eggplant	7.00 ± 1.49^ab^	4.40 ± 0.97^bc^	5.30 ± 0.82^ab^	3.90 ± 1.10^b^	6.90 ± 1.10^a^	20.50 ± 1.51^ab^	2.8 ± 0.79^ab^
Chilli	7.50 ± 1.27^a^	4.50 ± 0.53^bc^	2.40 ± 0.52^f^	4.30 ± 0.82^b^	6.70 ± 0.82^a^	17.90 ± 1.73^bc^	2.2 ± 0.42^b^
Mulberry	5.90 ± 1.20^ab^	3.80 ± 1.14^c^	2.20 ± 0.42^f^	3.20 ± 0.79^b^	6.70 ± 1.16^a^	15.90 ± 2.33^c^	2.4 ± 0.52^b^
Cotton	5.80 ± 1.14^ab^	5.10 ± 0.99^bc^	3.20 ± 1.14^def^	4.50 ± 0.97^b^	6.80 ± 1.23^a^	19.60 ± 3.34^ab^	2.5 ± 0.71^b^
Guava	6.30 ± 1.64^ab^	4.60 ± 0.97^bc^	5.40 ± 0.70^a^	3.70 ± 1.25^b^	7.00 ± 1.05^a^	20.70 ± 1.77^ab^	2.5 ± 0.53^b^
Papaya	6.70 ± 1.34^ab^	6.50 ± 1.27^a^	4.10 ± 0.88^cde^	3.80 ± 1.23^b^	7.40 ± 1.96^a^	21.80 ± 3.43^a^	2.5 ± 0.53^b^
Banana	7.10 ± 1.10^ab^	4.20 ± 0.92^bc^	4.20 ± 0.79^bcd^	6.10 ± 0.99^a^	7.90 ± 0.88^a^	22.40 ± 2.63^a^	2.4 ± 0.52^b^
Acalypha	6.30 ± 1.49^ab^	2.30 ± 0.48^d^	3.10 ± 0.74^def^	4.40 ± 0.52^b^	8.00 ± 2.31^a^	17.80 ± 3.46^bc^	3.4 ± 0.84^a^
Indian beech	7.70 ± 1.34^a^	5.20 ± 0.79^ab^	3.00 ± 0.82^ef^	4.10 ± 1.10^b^	6.90 ± 1.85^a^	19.20 ± 2.25^abc^	2.8 ± 0.63^ab^
df	9, 90	9, 90	9, 90	9, 90	9, 90	9, 90	9, 90
*F* value	3.16	14.01	22.62	6.11	1.47	6.06	3.04
*P* value	0.002**	< 0.001	< 0.001	< 0.001	0.171ns	< 0.001	0.003**
Eta squared (η^2^)	0.240	0.584	0.694	0.379	0.128	0.377	0.233

^#^Within each column, means followed by different superscripted lowercase letters differ significantly according to Tukey’s HSD test at *P* ≤ 0.01. ns = non-significant; ** = significant at 1% level.

The total nymphal period (1st–4th instars), which includes the duration between successive moults, showed a significant difference among host plants (*F* = 6.06; df = 9, 90; *P* < 0.001; η^2^ =0.377). The longest nymphal period was observed on banana (22.4 ± 2.63 days) and the shortest on mulberry (15.9 ± 2.33 days). The pupal period varied significantly (*F* = 3.04; df = 9, 90; *P* = 0.003**; η^2^ = 0.233), ranging from the longest on Acalypha (3.4 ± 0.84 days) to the shortest on chilli (2.2 ± 0.42 days).

**Fig. 1:**
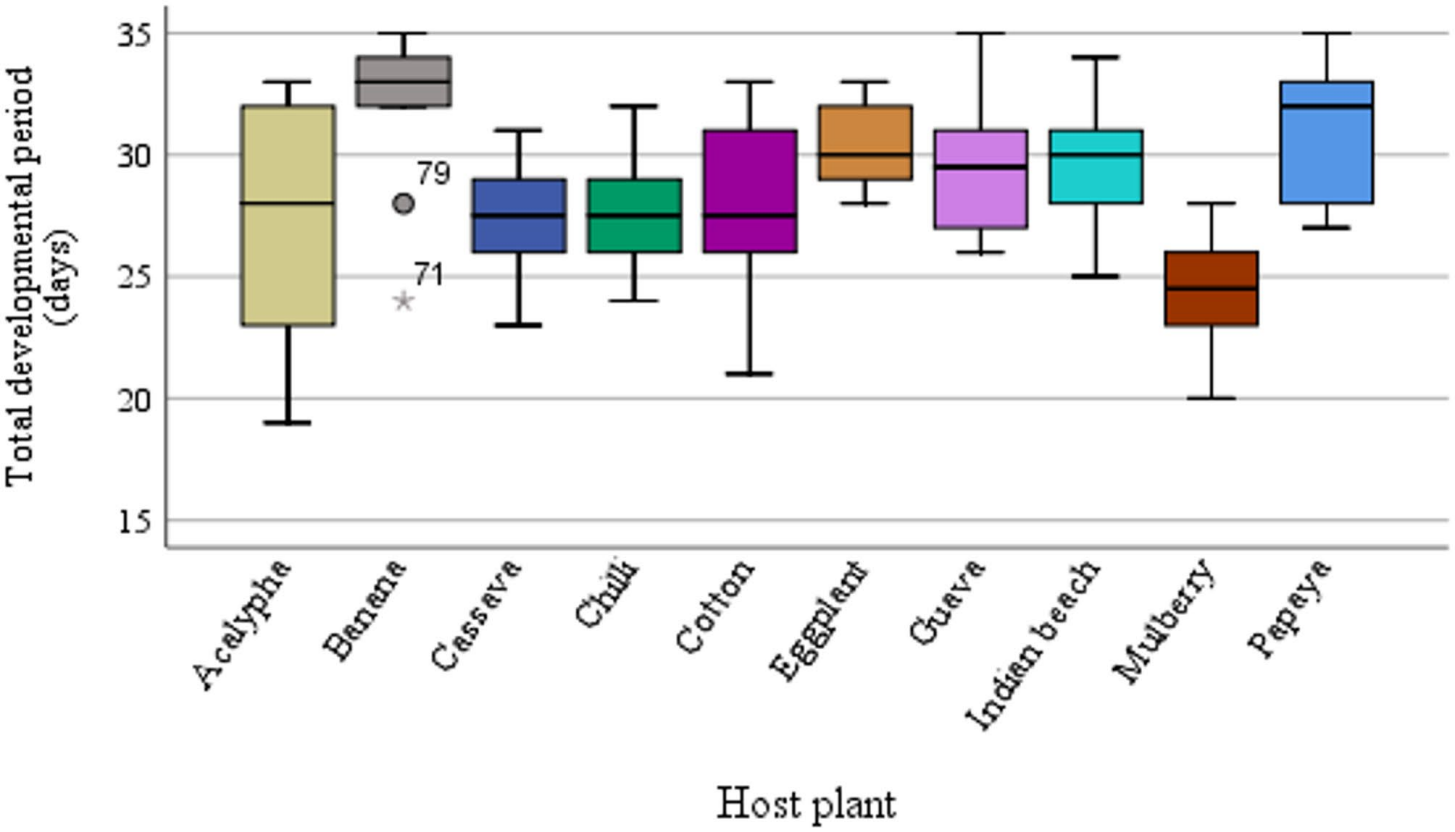
Total developmental period (days) of *Aleurodicus dispersus* across different host plants (*n* = 100 individuals total; *n* = 10 individuals per biological replicate) (*F* = 5.62; df = 9, 90; *P* < 0.001; η^2^ = 0.360). In each boxplot, the bold horizontal line represents the median, the box indicates the interquartile range (IQR), and the whiskers extend to 1.5× IQR above the third quartile and below the first quartile. Black dots represent outliers. The numbers 79 and 71 indicated in the figure are individual case identifiers (observation IDs) automatically assigned by the statistical software to highlight specific outlier data points. These represent individuals with atypically long developmental durations on banana and guava, respectively.

The TDP was significantly influenced by host plant (*F* = 6.06; df = 9, 90; *P* < 0.001; η^2^ = 0.360). The longest development was recorded on banana (31.9 ± 3.35 days), while the shortest was on mulberry (24.2 ± 2.39 days). The boxplot demonstrates a clear distinction in the median developmental periods for each host, reflecting the influence of host plant species on the insect’s life cycle (Fig. 1). The values 79 and 71 indicated in Fig. 1 represent the specific survival percentages of the cohort on the highest and lowest performing hosts, respectively. Cassava, mulberry, and cotton exhibited shorter TDPs, with median values positioned towards the lower end of the interquartile range (IQR). Banana and papaya displayed the longest TDPs, indicating a slower developmental process. Host plants such as chilli and guava showed moderate developmental durations, with relatively smaller IQRs suggesting less variability in development time among replicates. Mortality rates were notably higher on hosts with extended TDPs, such as banana and Indian beech.

### Reproductive parameters across host plants

**Fig. 2:**
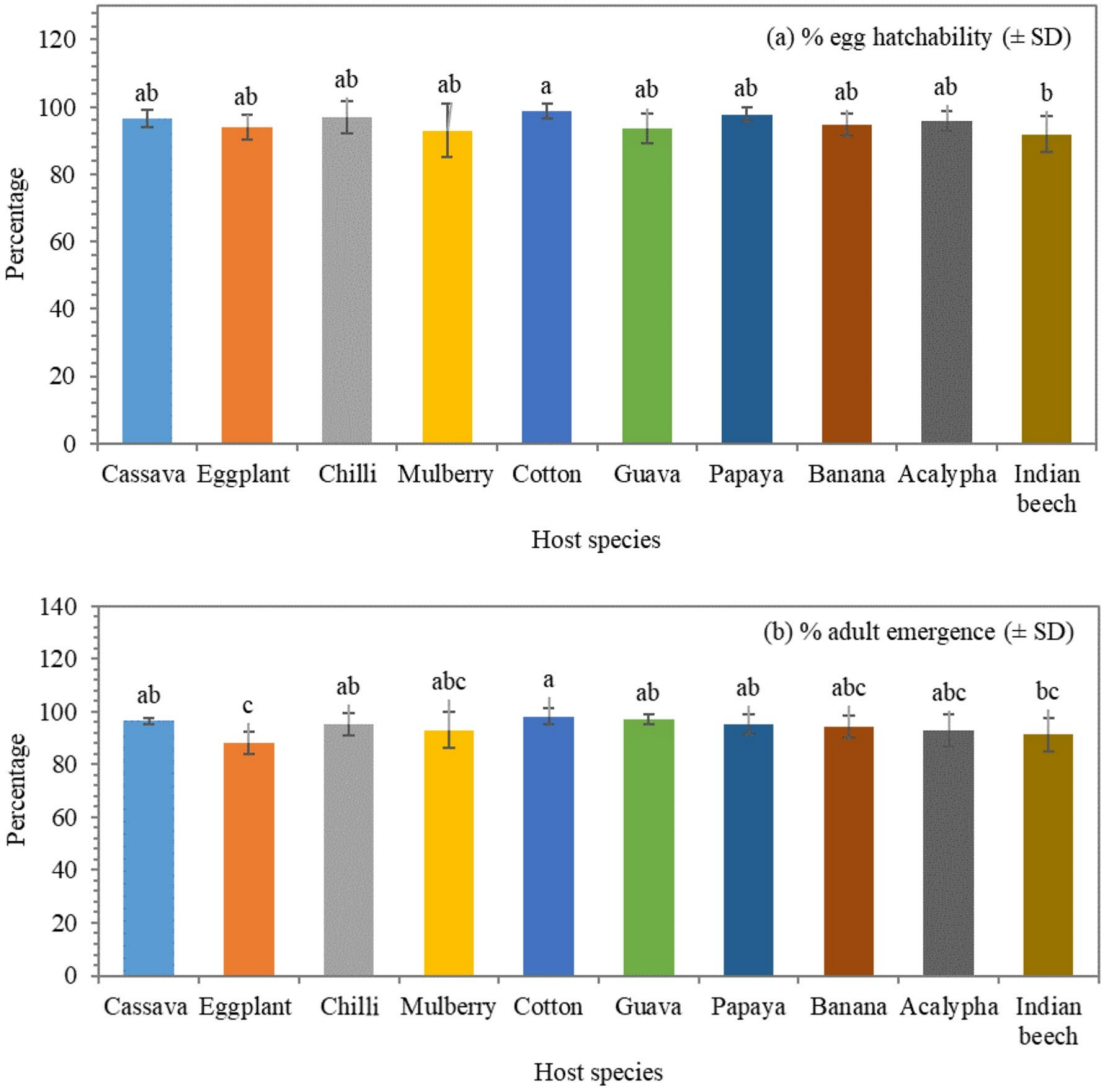
(**a**) Percent egg hatchability (*F* = 2.67; df = 9, 90; *P* = 0.009**; η^2^ = 0.211) and (**b**) percent adult emergence (*F* = 4.17; df = 9, 90; *P* < 0.001; η^2^ = 0.294) of *Aleurodicus dispersus* across different host plants. Bars represent mean ± standard deviation (*n* = 100 individuals total; *n* = 10 individuals per biological replicate). Different letters indicate statistically significant differences among host plants based on Tukey’s post hoc test at *P* < 0.01. Stage-specific mortality during the egg-to-nymph transition was highest on eggplant and Indian beech.

Egg hatchability varied significantly among host plants (*F* = 2.67; df = 9, 90; *P* = 0.009**; η^2^ = 0.211) (Fig. 2a). The highest hatchability was observed on cotton (98.7 ± 9.9%), and the lowest on Indian beech (91.9 ± 9.6%). Unsuccessful hatches and subsequent mortality in the first instar stage were most frequent on eggplant and Indian beech. Similarly, adult emergence showed significant variation (*F* = 4.17; df = 9, 90; *P* < 0.001; η^2^ = 0.294). The highest percentage was recorded on cotton (98.2 ± 9.9%), while eggplant (88.4 ± 9.4%) supported the lowest emergence (Fig. 2b).

**Fig. 3:**
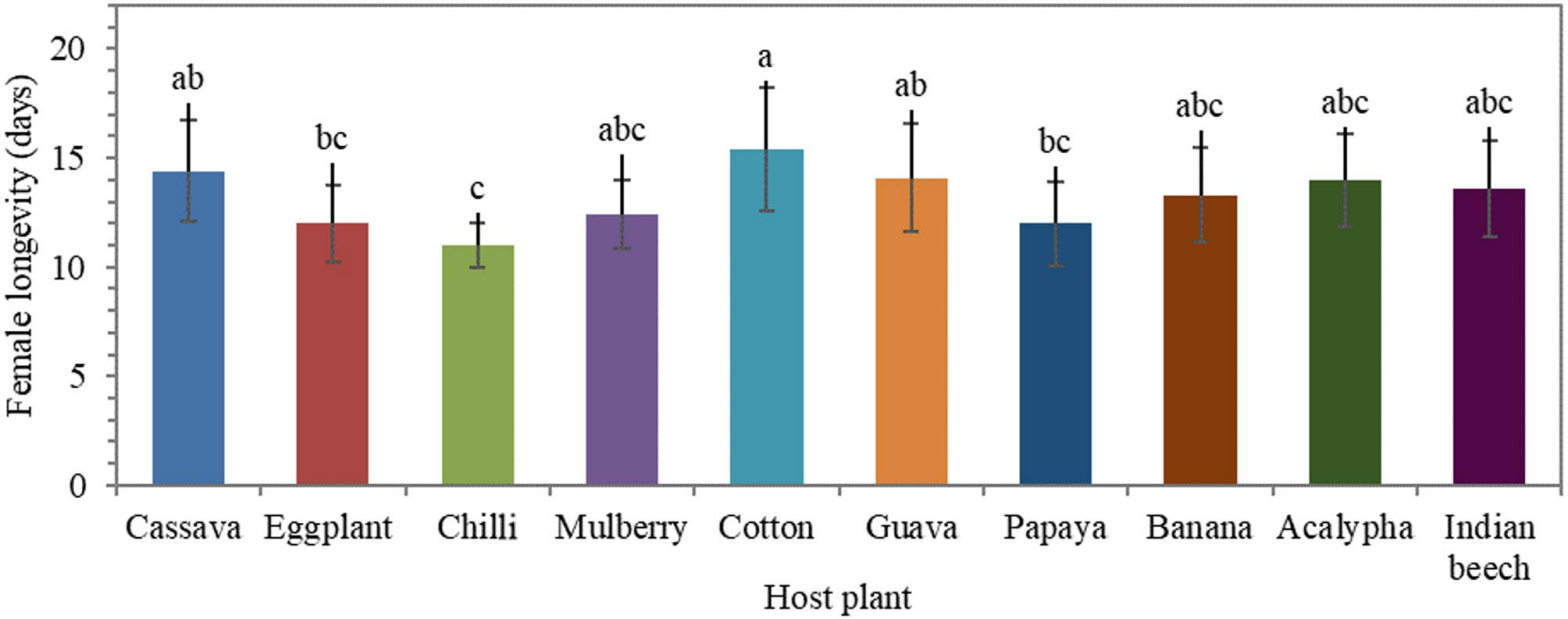
Female longevity (days) of *Aleurodicus dispersus* across different host plants (*F* = 4.12; df = 9, 90; *P* < 0.001; η^2^ = 0.292). Bars represent mean ± standard deviation (*n* = 100 individuals total; *n* = 10 individuals per biological replicate). Different letters indicate significant differences among host plants based on Tukey’s post hoc test at *P* < 0.01. The pre-oviposition period remained constant at approximately 0.5 days across all host plants.

**Fig. 4:**
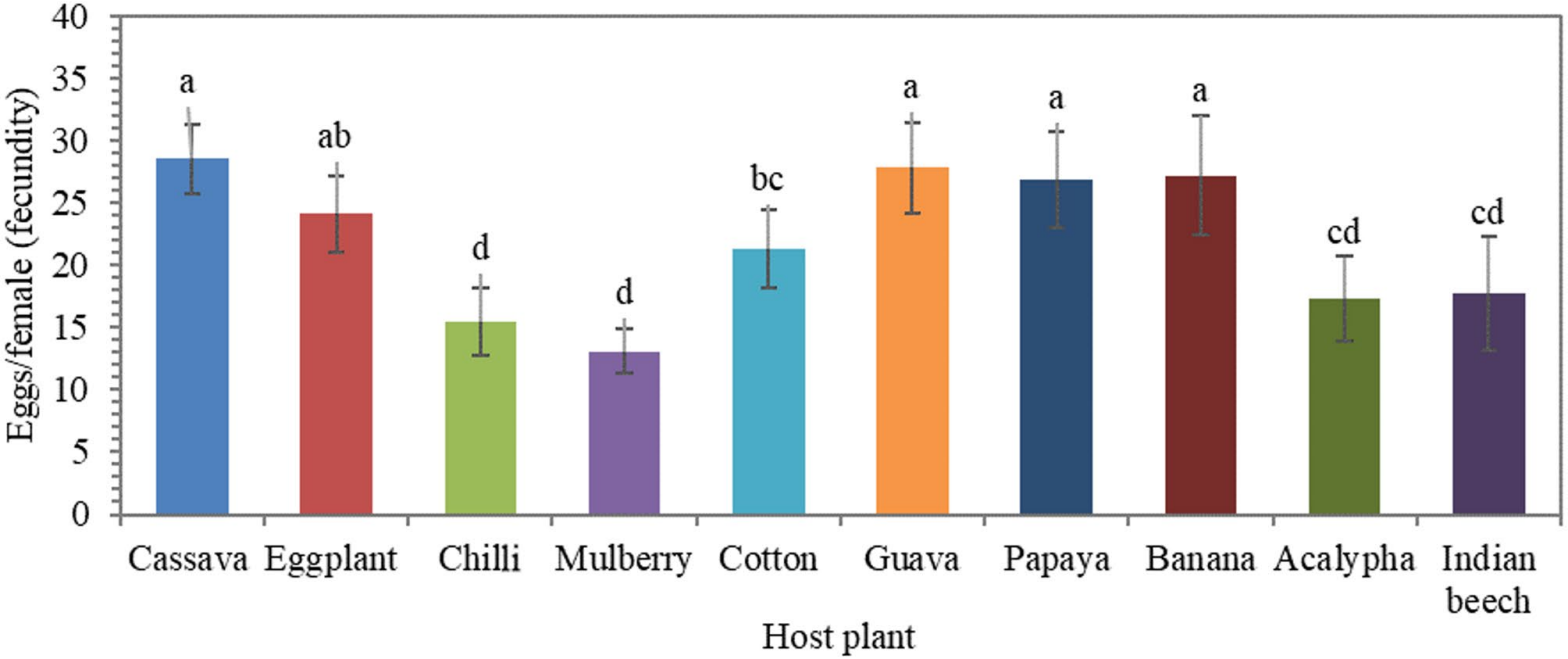
Fecundity (eggs per female) of *Aleurodicus dispersus* across different host plants (*F* = 27.20; df = 9, 90; *P* < 0.001; η^2^ = 0.731). Bars represent mean ± standard deviation (*n* = 100 individuals total; *n* = 10 individuals per biological replicate). Different letters indicate significant differences among host plants based on Tukey’s post hoc test at *P* < 0.01.

Female adult longevity was significantly affected by host plant (*F* = 4.12; df = 9, 90; *P* < 0.001; η^2^ = 0.292) (Fig. 3). The longest lifespan was observed on cotton (15.4 ± 2.84 days), followed by cassava (14.4 ± 2.32 days), whereas the shortest was on chilli (11.0 ± 1.05 days). The pre-oviposition period remained statistically similar across all host plants, with an average of approximately 0.5 days. Fecundity showed highly significant differences across host species (*F* = 27.20; df = 9, 90; *P* < 0.001; η^2^ = 0.731) (Fig. 4). The highest fecundity was recorded on cassava (28.5 ± 3.72 eggs/female), followed by guava (27.8 eggs/female), while the lowest was observed on mulberry (13.1 ± 1.79 eggs/female).

The sex ratio (female: male) was significantly affected by host plant (*χ*^2^ = 42.124, df = 9, *P* < 0.001) (Table 3). Progeny populations were noticeably more female-biased on cotton (1:0.27) and eggplant (1:0.37) (Table 4). In contrast, a male-biased distribution was observed on chilli (1:0.85), guava (1:1.79), and mulberry (1:1.36). These results indicate that host plant quality and species significantly favour different sex allocations in *A. dispersus* populations. The strength of association between host plants and sex ratio (Cramer’s V = 0.262) suggests a moderate association.

**Table 3:** Chi-square test results and symmetric measures of sex ratio distribution of *Aleurodicus dispersus* across different host plants.

a. Chi-square tests						
Particulars	Value		Degrees of freedom (df)		Asymptotic significance (2-sided)	
Pearson chi-square	42.124^a^		9		0.000	
Likelihood ratio	42.758		9		0.000	
Number of valid cases	614					

b. Symmetric measures			
Particulars		Value	Approximate significance
Nominal by nominal	Phi	0.262	0.000
	Cramer’s V	0.262	0.000
Number of valid cases		614	

^a^All cells had expected counts greater than 5; the minimum expected count was 10.59.

**Table 4:** Distribution of female and male *Aleurodicus dispersus* individuals across different host plants (*n* = 20 plants per host plant).

Host plant	Female count	Male count	Total
Acalypha	20	10	30
Banana	63	50	113
Cassava	71	34	105
Chilli	20	17	37
Cotton	48	13	61
Eggplant	19	7	26
Guava	24	43	67
Indian beech	31	21	52
Mulberry	28	38	66
Papaya	40	17	57
Total	364	250	614

The sex ratio is calculated as (Number of Males / Number of Females) to express the proportion of males for every one female. The counts represent the total number of adults that successfully emerged from the 20 plants monitored per host.

### Relationship between egg hatchability and adult emergence

**Fig. 5:**
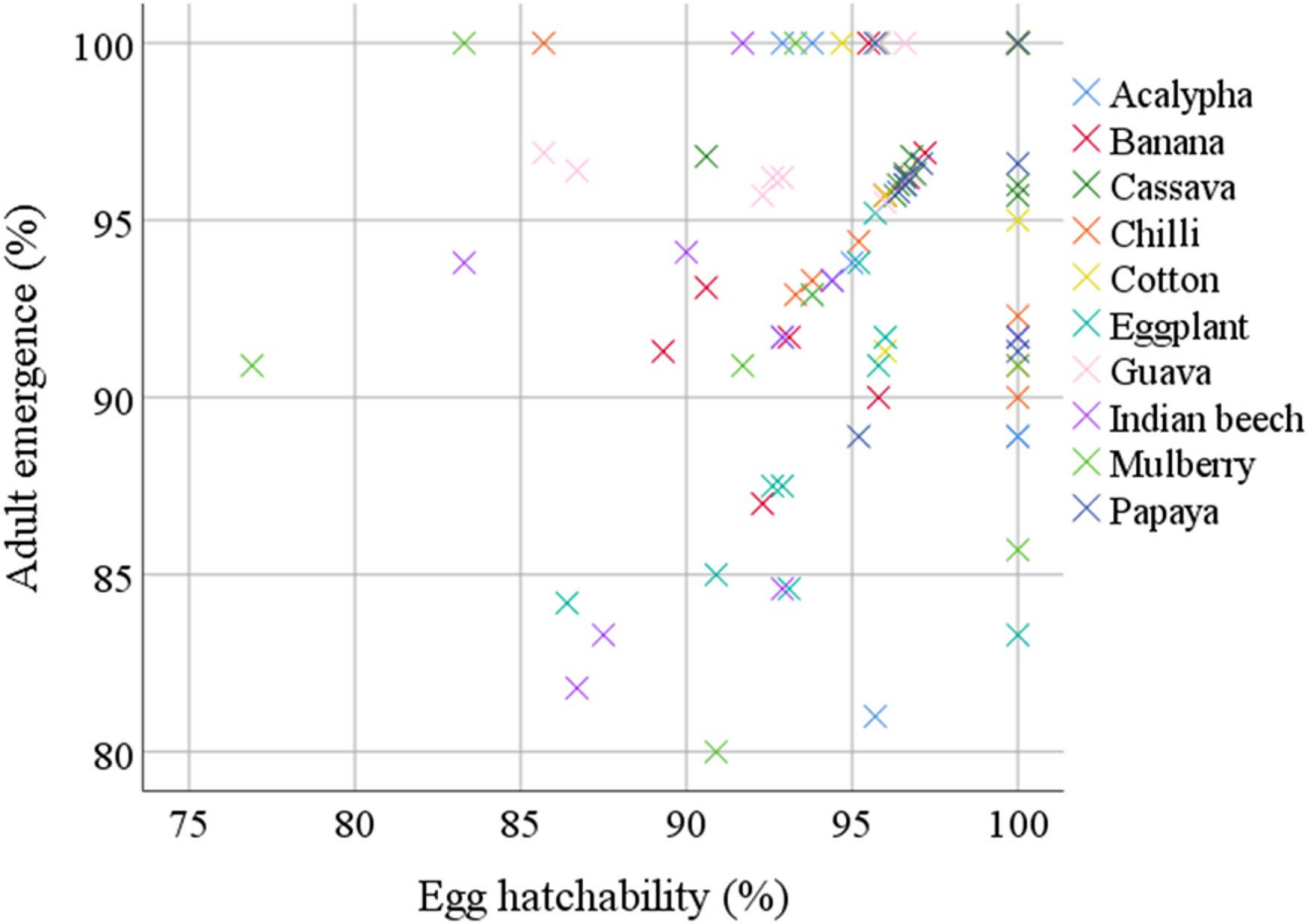
Linear relationship between egg hatchability (%) and adult emergence (%) of *Aleurodicus dispersus* across different host plants. A strong positive correlation (*r* = 0.85; *P* < 0.001) indicates that host plants supporting high egg viability also facilitate successful moult to adulthood. This relationship is described by the linear regression equation Y = 12.5 + 0.83X (*R*^2^ = 0.72), with a coefficient of determination (*R*^2^ = 0.72), implying that approximately 72% of the variation in adult emergence is explained by egg hatchability. Y = adult emergence (%); X = egg hatchability (%).

A statistically significant and strong positive relationship was observed between egg hatchability and adult emergence across the host plants tested (Fig. 5). The Pearson correlation coefficient (*r* = 0.85; *P* < 0.001) indicated a highly positive correlation, suggesting that host plants providing a favourable environment for egg viability also facilitated successful development through to the adult stage (Table 5). The regression analysis revealed the equation Y = 12.5 + 0.83X, where Y represents adult emergence (%) and X represents egg hatchability (%). The coefficient of determination (*R*^2^ = 0.72) indicated that 72% of the variation in adult emergence was explained by egg hatchability.

**Table 5:** Summary of linear regression relationships between key biological parameters of *Aleurodicus dispersus*.

S. no.	Relationship	Regression equation	Correlation coefficient (*r*)	*R* ^2^	Significance (*P* value)
1	Adult emergence vs. egg hatchability	Y = 12.5 + 0.83X	0.85	0.72	< 0.001
2	Fecundity vs. total developmental period	Y = 125.4 − 3.21X	-0.78	0.61	< 0.001

All relationships were statistically significant at the 0.1% level, indicating strong associations. *Y* = dependent variable (response); *X* = independent variable (predictor).

### Relationship between total developmental period and fecundity

**Fig. 6:**
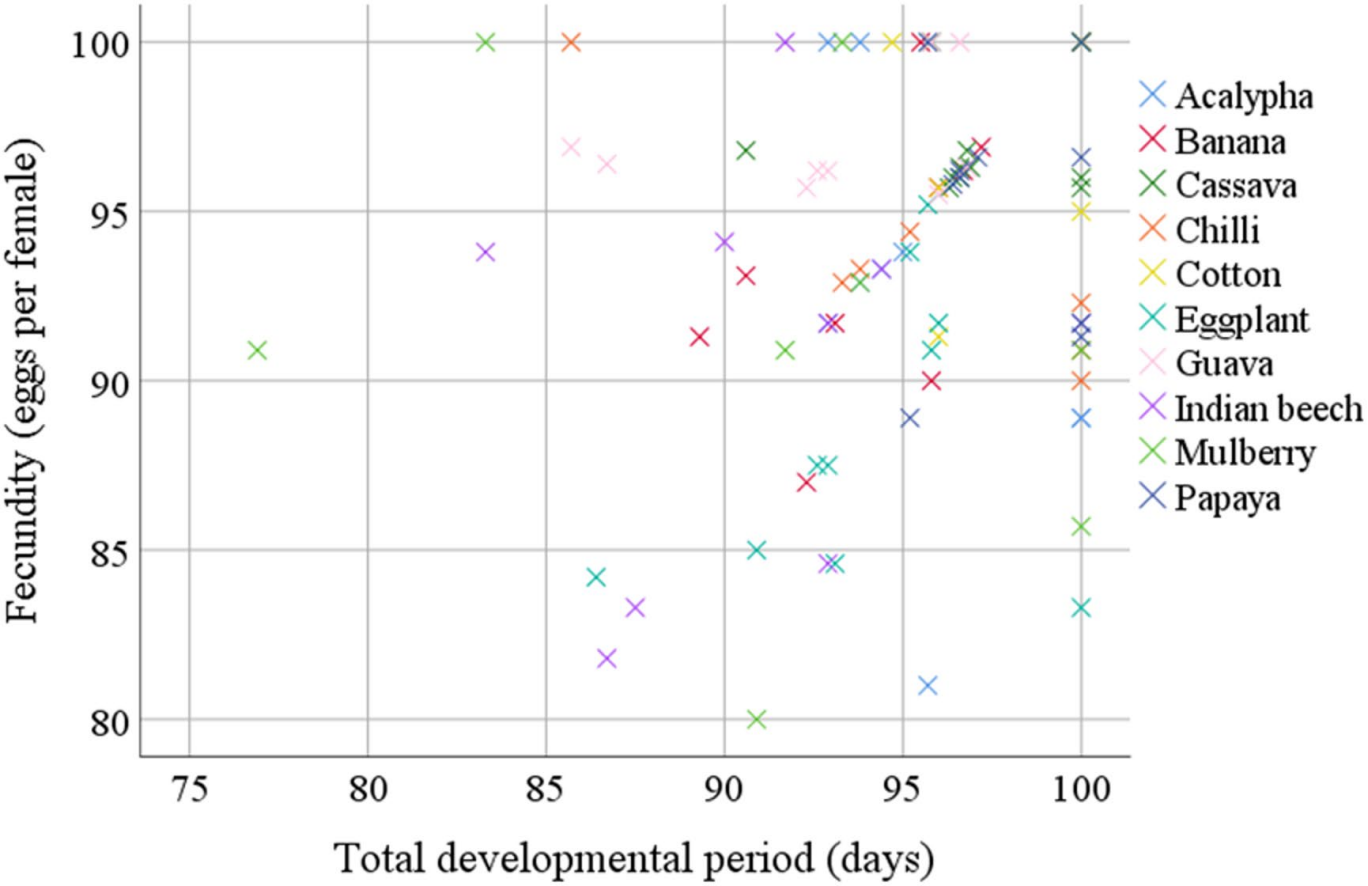
Linear relationship between total development period (days) and fecundity (eggs per female) of *Aleurodicus dispersus* across different host plants. The Pearson correlation coefficient (*r* = − 0.78; *P* < 0.001) indicates a strong inverse relationship, confirming that less favourable host plants result in delayed maturity and significantly reduced reproductive output. The association is described by the linear regression equation Y = 125.4 − 3.21X (*R*^2^ = 0.61), with a coefficient of determination (*R*^2^ = 0.61), implying that approximately 61% of the variation in fecundity is explained by the total developmental duration. Y = fecundity (eggs per female); X = total developmental period (days).

A significant negative correlation was found between the total developmental period (TDP) and fecundity of *A. dispersus* across the ten host plants evaluated (Fig. 6). The Pearson correlation coefficient (*r* = − 0.78; *P* < 0.001) indicated a strong inverse relationship, suggesting that longer developmental durations often caused by less favourable host quality were associated with reduced reproductive output (Table 5). The linear regression equation derived from the data was Y = 125.4 − 3.21X, where Y represents fecundity (eggs per female) and X is the total developmental period (days). The coefficient of determination (*R*^2^ = 0.61) revealed that approximately 61% of the variation in fecundity was explained by differences in developmental duration.

## Discussion

The findings of this study clearly demonstrate that host plant species exert significant influence on the developmental biology and reproductive performance of *A. dispersus*. The observed variations in developmental duration, fecundity, egg hatchability, adult emergence, adult longevity, and sex ratio are consistent with the patterns noted in earlier studies on both *A. dispersus *and related whitefly species, particularly *B. tabaci*, highlighting the critical role of host plant quality in shaping pest life-history traits.

The significantly shorter total developmental period (TDP) recorded on cassava and mulberry, and the prolonged duration on banana and Indian beech, aligns with earlier findings by Sampiano and Aceres^12^. They noted that *A. dispersus* completed its development more quickly on cassava and guava due to favourable leaf characteristics such as a thinner epidermis and soft tissues. These characteristics likely enhance feeding efficiency and reduce the energetic cost of development. The process of successive moults from the first to fourth instar was notably faster on these hosts. Similarly, Kedar et al.^10^ observed faster development of *B. tabaci* on nutrient-rich hosts like cotton and tomato, reinforcing the idea that high nitrogen content in foliage can accelerate whitefly development. Our results also corroborate the findings of Al-Zeyoud and Sengonca^13^, who observed shorter development periods due to improved nutritional compatibility.

Egg hatchability was highest on cotton and cassava, which supports earlier reports that hosts with smoother and softer leaves and favourable moisture conditions promote successful embryonic development^1^. Manoharan et al.^14^ similarly observed higher hatchability on cassava, attributing it to the thin cuticle and moist surface. In contrast, the lower hatchability and increased mortality of early instars on Indian beech and eggplant suggest that these hosts are less favourable, potentially due to mechanical or chemical defenses that hinder successful hatching and initial feeding^15^. Adult emergence followed a similar pattern, supporting the conclusions of Gamal^16^, who demonstrated that host palatability directly influence whitefly emergence rates. The strong positive correlation observed between egg hatchability and adult emergence (*r* = 0.85) further supports the hypothesis that early-stage success is critical for overall survival^17^.

The high η^2^ value (0.73) for fecundity suggests that host plant species explain over 70% of the variation in egg production. The highest egg-laying was observed on cassava, followed by guava and papaya, mirroring results by Anjum and Ahmed^11^ and Omondi et al.^18^. Conversely, the relatively low fecundity on eggplant and chilli may result from the presence of anti-feedant compounds^7^. The observed negative correlation between TDP and fecundity (*r* = − 0.78) aligns with earlier reports suggesting that faster development allows earlier and more frequent reproduction^9^.

Adult longevity was significantly greater on cotton, cassava, and guava. Longer adult lifespan may enhance pest population buildup because it extends the reproductive period^10,19^. The host-specific sex ratio (Female: Male) variations, with female-bias on guava and eggplant and male-bias on cotton and banana, suggest an influence of host quality on sex allocation. Huang et al.^20^ observed that nutrient-rich environments often favour the production of females.

The host-specific performance of *A. dispersus* observed under controlled conditions may have important ecological implications under field scenarios. Highly suitable hosts such as cassava and cotton may accelerate population growth and increase pest pressure^1,21^. However, interactions with natural enemies, including parasitoids (*Encarsia* spp., *Eretmocerus* spp.) and predators such as *Chrysoperla carnea*, may suppress whitefly populations under field conditions^2,22^. In addition, host plant defenses, including trichomes and allelochemicals, can reduce whitefly performance or enhance natural-enemy effectiveness^23,24^. Some plants may also emit volatile compounds that attract natural enemies^25^. Therefore, although cassava supported optimal development under laboratory conditions, its impact in the field may be moderated by biotic interactions. Poorer hosts like eggplant or Indian beech may function as ecological sinks or trap crops within integrated pest management systems^22^.

A acknowledged limitation of the present study is that it relied on cohort-based observations rather than a full age-stage, two-sex life table technique. While our current method clearly identifies host suitability, the age-stage, two-sex life table would be much better and more explainable as it accounts for the variable developmental rates of both sexes and overlapping generations^26,27^. Future research will incorporate this technique to provide a more refined understanding of population growth parameters (*r*, *λ*, *R*_0_) under these fluctuating environmental conditions.

## Conclusions

The findings from this study have important implications for host-targeted pest management strategies against *A. dispersus*. Host plants such as cassava, cotton, and guava, which supported relatively rapid development, higher egg hatchability, and greater fecundity, represent favourable reservoirs for whitefly multiplication. These crops should be monitored closely for early infestation. Conversely, crops such as Indian beech, eggplant, and banana, which exhibited comparatively lower whitefly performance, may be considered in cultural practices such as border cropping or trap cropping to reduce pest buildup. The strong correlations between egg hatchability, adult emergence, and the total developmental period provide useful indicators for forecasting potential pest outbreaks. Overall, the results contribute to a better understanding of host-mediated variation in the biology of *A. dispersus* and support the development of ecologically informed pest management strategies.

## Data Availability

Data is provided within the manuscript file.

## Abbreviations

*A. dispersus*: *Aleurodicus dispersus*
*B. tabaci*: *Bemisia tabaci*
CRD: Completely randomized design
IQR: Interquartile range
SD: Standard deviation
IBM: International business machines
NY: New York
USA: United States of America
F: F value
df: Degrees of freedom
P: P value
IPM: Integrated pest management
Ns: Non-significant
r: Correlation coefficient
Y: Dependent variable
X: Independent variable
TDP: Total developmental period
ANOVA: Analysis of variance
HSD: Honest significant difference
R^2^: Coefficient of determination
η^2^: Eta-squared
n: Number
TNAU: Tamil Nadu Agricultural University
PKM: Periyakulam
MR: Mildew Resistant
Co: Coimbatore
TRY: Trichy
ICAR-IARI: Indian Council of Agricultural Research-Indian Agricultural Research Institute
cm: Centimeter
h: Hour
χ^2^: Chi-squared
R_0_: Net reproductive rate (offspring/individual)
*r*: Intrinsic rate of increase (day^–1^)
*λ*: Finite rate of increase (day^–1^)
